# Stair-Climbing Wheeled Robot Based on Rotating Locomotion of Curved-Spoke Legs

**DOI:** 10.3390/biomimetics9100633

**Published:** 2024-10-17

**Authors:** Dongwoo Seo, Jaeyoung Kang

**Affiliations:** Department of Mechanical Engineering, Inha University, Incheon 22212, Republic of Korea

**Keywords:** stair climbing, wheel climbing, curved spoke, wheel legged, locomotion

## Abstract

This study proposes a new wheel-leg mechanism concept and formulations for the kinematics and dynamics of a stair-climbing robot utilizing the rotating leg locomotion of curved spokes and rolling tires. The system consists of four motor-driven tires and four curved-spoke legs. The curved-spoke leg is semicircle-like and is used to climb stairs. Once the spoke leg rolls on the surface, it lifts and pulls the mating wheel toward the surface, owing to the kinematic constraint between the spoke and the wheel. Single-wheel climbing is a necessary condition for the stair climbing of whole robots equipped with front and rear axles. This study proposes the design requirements of a spoke leg for the success of single-wheel climbing in terms of kinematic inequality equations according to the scenario of single-wheel climbing. For a design configuration that enables single-wheel climbing, the required minimum friction coefficient for the static analysis of the stair-climbing wheeled robots is demon-strated. Thereafter, the stair-climbing ability is validated through the dynamic equations that enable the frictional slip of the tires, as well as the curved-spoke legs. Lastly, the results revealed that the rotating locomotion of the well-designed curved-spoke legs effectively enables the stair climbing of the whole robot.

## 1. Introduction

Stair climbing is a very challenging task for an autonomous robot, and stair-climbing robots have been studied over decades through several types of platforms, such as wheelchairs, robots, and ground robots. These are often categorized based on their own locomotion mechanisms, and review articles have categorized these into four or five, including tracked, legged, wheel-linkage, and wheel-legged platforms [1,2,3,4]. The track-based mechanism enables the effective up-and-down climbing of robots [5,6,7,8] and may be less sensitive to the size and shape of a stair. Tracked robots are sometimes equipped with several track modules or flippers to overcome the slip problem of tracks during stair climbing; however, tracks are known to be inefficient for driving on normal flat road.

If well-equipped with sensors and actuators, legged robots can demonstrate excellent stair-climbing performance, but they require a highly complicated control scheme and hardware [9,10,11,12,13,14,15,16]. Particularly, they require a significant degree of freedom to mimic the locomotion of animals and humans, which can effectively achieve stair climbing. Two-legged, four-legged, and even six-legged robots have been reported to demonstrate excellent stair-climbing performance. It is well known that rolling wheels are the most effective mechanism for transport on a flat road. Therefore, the combination of efficient and simple stair-climbing mechanisms and rolling wheels is still highly recommended in autonomous mobile platforms for both indoor and outdoor uses.

The wheel-linkage mechanism can allow for the high efficiency required for driving on a flat surface and for climbing stairs [17,18,19,20,21,22,23]. Typically, this platform lifts each wheel to the higher surface of the stair by the articulation of linkages or the rotation of wheel clusters [17,18,19,20,21]. An example of this mechanism is the rocker–bogie mechanism [22,23], which utilizes the friction of the rolling wheel on the riser of a stair, making it impossible to climb the stair without the riser. Generally, one drawback of this mechanism is the relatively low stair-climbing speed owing to the slow speed of the linkage actuators.

The wheel-leg mechanism is a prominent mechanism among the locomotion mechanisms, owing to its good stair-climbing ability and relatively lower complexity [24,25,26,27,28,29,30,31,32,33,34,35,36,37]. Examples include the curved spoke and starfish-like spoke, which are designed for legs to rotate on and climb a stair. The curved leg rotates and touches down on the higher surface of the stair and lifts the whole body by rolling on the surface. A well-defined design of the size and geometry of the spoke is crucial to the success of stair climbing on stairs with various shapes and sizes.

In this study, we developed a new design mechanism, and its design strategy was based on the form of simple wheel-leg mechanisms, which are efficient in stair climbing and driving on flat road. The design was equipped only with in-wheel motor-driven tires and curved-spoke legs, which are significantly less complex in terms of system design and implementation. However, the main issue with this system is the design of the configuration of the motor-driven wheel and the curved-spoke leg for efficient stair climbing on stairs with various geometries.

Therefore, we present both kinematic and dynamic models to demonstrate the feasibility of the system for stair-climbing missions. To this end, first, we derived the kinematic constraint equations for a single wheel to climb the stair using the curved-spoke leg and broadly investigated the success of the kinematic model for stair climbing as a function of the stair geometry, such as the width and height of a stair. To validate the design configuration based on the kinematic equations of single-wheel climbing, the dynamic equations of motion were derived for the entire robot equipped with four in-wheel motor-driven tires and curved-spoke legs. Thereafter, the dynamic stair-climbing simulation of the entire system was demonstrated, such that the front and rear axles of the robot can effectively climb stairs even with the slips of tire and spoke legs if the design configuration is well defined.

## 2. Materials and Methods

**Figure 1:**
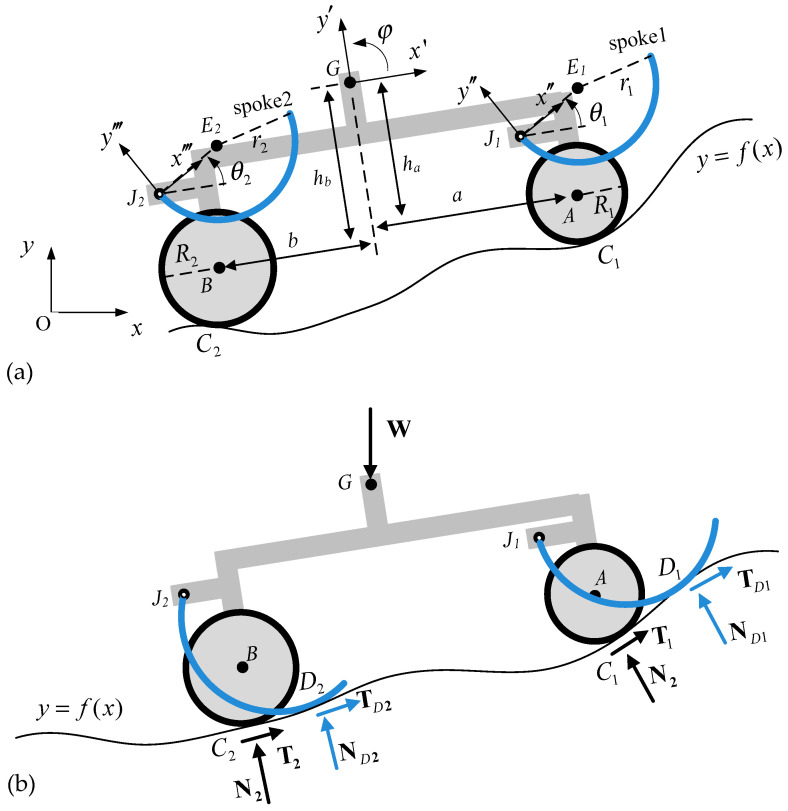
Robot with motor-driven tires and rotating curved-spoke legs; (**a**) configuration of the system; (**b**) free body diagram.

One of the main design goals of the proposed robot was a high driving performance on an outdoor flat road. Therefore, the center of gravity (CG) was set to be the geometric center between the front and rear axles for a higher lateral driving stability. In addition, the robot consisted of four motor-driven tires and corresponding curved-spoke legs near them, as seen in Figure 1a. This design was simplified into a two-dimensional plane motion model as shown in Figure 1 by assuming that it climbs the left and right symmetrical stairs and that the left and right actions are also symmetric. The curved-spoke legs were rotated by motors attached to spoke joints and generated torque to push and roll on the higher surface and bring the nearby wheels to the surface if necessary. Therefore, the role of the curved-spoke leg was to assist the nearby wheel with climbing the obstacle until the wheel rode on it. We do not presently classify this conceptual robot as either a legged or wheeled stair-climbing robot. In the end, however, it will turn out to be one of them, depending on the design constraints in terms of the friction coefficient required for stair climbing.

The equations of motion (EOMs) derived for the system with curved-spoke legs pushing the surface, as shown in Figure 1b, can be expressed as follows:

(1) $$\begin{array}{cc} m\ddot{x}i+m\ddot{y}j & =N_{1}+T_{1}+N_{2}+T_{2}+N_{D1}+T_{D1}+N_{D2}\\ & +T_{D2}-mgj \end{array},$$

(2) $$\begin{array}{cc} I_{G}\dot{\omega } & =r_{C_{1}/G}\times \left(N_{1}+T_{1}\right)+r_{C_{2}/G}\times \left(N_{2}+T_{2}\right)\\ & +r_{D_{1}/G}\times \left(N_{D1}+T_{D1}\right)+r_{D_{2}/G}\times \left(N_{D2}+T_{D2}\right) \end{array},$$

where i, j, and k are the direction vectors in the x, y, and z axes, $r_{C/G}$ denotes the position vector from position C to position G, and $\omega =\dot{\varphi }k$.

**Figure 2:**
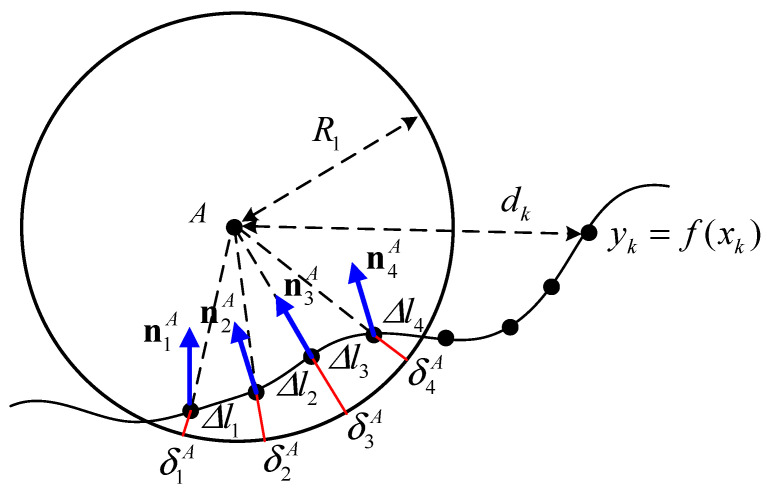
Contact penalty method for tire A.

In the EOM, the tire contact forces $N_{j}$ and $T_{j}$ are the normal and traction forces, respectively, acting on the surface profile, which is discretized by a continuous function $y_{k}=f(x_{k})$, as illustrated in Figure 2. To measure the deflection and the speed of the contact point of tire A, the distance function between the wheel center A and the discretized contact point is defined as

(3) $$d_{k}^{A}={\left\{{\left(x_{A}-x_{k}^{A}\right)}^{2}+{\left(y_{A}-f(x_{k}^{A})\right)}^{2}\right\}}^{1/2},$$

subject to

$$j=\left\{\left.k\right|d_{A}^{k}<R_{1}\right\},k=1,2,\cdots ,N_{g},$$

where $N_{g}$ is the total number of discretized points of the surface profile function.

The contact nodal normal and tangential loads at a discretized contact node of tires A and B are defined in the combination of the linear spring and damping elements in terms of the contact radial deflection δ as

(4) $$N_{j}\equiv N_{j}n_{j}=\frac{1}{2}\left({\bar{N}}_{j}+\left|{\bar{N}}_{j}\right|\right)n_{j},$$

(5) $${\bar{N}}_{j}=\left(k_{c}\Delta l_{j}\delta_{j}+c_{c}\Delta l_{j}{\dot{\delta }}_{j}\right)n_{j}\cdot l_{j},$$

(6) $$T_{j}=\mu N_{j}t_{j},$$

where the tangential stiffness load is neglected, $t_{j}$ is the unit tangential vector at the j-th contact node, $l_{j}$ is the unit direction vector between the tire center and the j-th contact node, $k_{c}$ and $c_{c}$ are the linear contact stiffness and damping constant per unit contact length of the tire, μ is the friction coefficient, and

(7) $$\delta_{j}=R-d_{j},$$

(8) $$\Delta l_{j}={\left\{{\left(x_{j+1}-x_{j}\right)}^{2}+{\left(f_{j+1}-f_{j}\right)}^{2}\right\}}^{1/2},$$

(9) $${\dot{\delta }}_{j}=-\left(\omega \times r_{A/G}+{\dot{r}}_{G}\right)\cdot n_{j},$$

**Figure 3:**
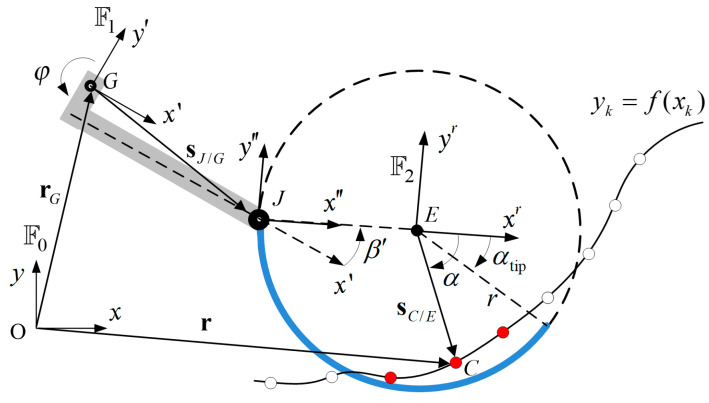
Contact penalty method for the contact forces of the curved-spoke leg; $F_{0}$, $F_{1}$, and $F_{2}$ denote coordinate system xyz (global), x′y′z′ (robot body-fixed), and x″y″z″ (spoke-fixed), respectively.

The contact loads of the curved-spoke leg are also defined in the normal and tangential directions when the part of the spoke is in contact with the surface profile, as shown in Figure 3. For this, the position vector of contact node C is described in the given coordinate systems as

(10) $$r=\left[\begin{array}{c} x\\ y \end{array}\right]=r_{G}+R_{1}^{0}s_{J/G}^{\prime }+R_{2}^{0}s_{E/J}^{\prime \prime }+r_{C/E},$$

where s, s′, s″, and $s^{r}$ denote the vector described in the global xyz, robot body-fixed x′y′z′, spoke-fixed J-origin x″y″z”, and spoke-fixed E-origin $x^{r}y^{r}z^{r}$ coordinates, respectively, and

(11) $$r_{C/E}=R_{2}^{0}s_{C/E}^{r}=R_{2}^{0}\left[\begin{array}{c} x^{r}\\ y^{r} \end{array}\right],$$

(12) $$s_{J/G}^{\prime }=\left[\begin{array}{c} a-L_{1}\\ -h_{G}-(h_{A}-h_{1}) \end{array}\right],$$

(13) $$s_{E/J}^{\prime \prime }=\left[\begin{array}{c} r_{1}\\ 0 \end{array}\right],$$

where $R_{m}^{I}$ is the transformation matrix by rotation from the *I*-th to the *m*-th coordinate defined in Figure 3. Therefore, the contact nodal position C can be expressed in the spoke-fixed E-origin $x^{r}y^{r}z^{r}$ coordinate as

(14) $$\begin{array}{cc} s_{C/E}^{r} & =\left[\begin{array}{c} x^{r}\\ y^{r} \end{array}\right]\\ & ={\left(R_{2}^{0}\right)}^{T}\left\{\left[\begin{array}{c} x\\ y \end{array}\right]-\left(r_{G}+R_{1}^{0}s_{J/G}^{\prime }+R_{2}^{0}s_{E/J}^{\prime \prime }\right)\right\} \end{array}$$

Next, the j-th contact nodes shown in the red dot in Figure 3 are defined if the discretized nodes of the surface profile are within the contact regime of the curved-spoke leg, satisfying the following two conditions as

(15) $$j=j^{\left(1\right)}\cap j^{\left(2\right)},$$

where

$$\left\{\begin{array}{l} j^{\left(1\right)}=\left\{\left.k\right|{\left(x_{k}-x_{E}\right)}^{2}+{\left(f_{k}-y_{E}\right)}^{2}<r_{1}\right\}\\ j^{\left(2\right)}=\left\{\left.k\right|-\pi <\tan^{-1}\left(\frac{y^{r}(x_{k},f_{k})}{x^{r}(x_{k},f_{k})}\right)<\alpha_{\mathrm{tip}}\right\} \end{array}\right.,$$

and where $k=1,2,\cdots ,N_{g}$. Consequently, the corresponding contact nodal normal and tangential loads at the detected j-th contact node of the curved-spoke leg are obtained using the same method used to calculate the tire contact loads:

(16) $$N_{j}^{D}\equiv N_{j}^{D}n_{j}^{D}=\frac{1}{2}\left({\bar{N}}_{j}^{D}+\left|{\bar{N}}_{j}^{D}\right|\right)n_{j}^{D},$$

(17) $${\bar{N}}_{j}^{D}=\left(k_{D}\Delta l_{j}^{D}\delta_{j}^{D}+c_{D}\Delta l_{j}^{D}{\dot{\delta }}_{j}^{D}\right)n_{j}^{D}\cdot l_{j}^{D},$$

(18) $$T_{j}^{D}=\mu_{D}N_{j}^{D}t_{j}^{D},$$

where the scalar and vector notations are the same as the tire contact model, and the sub- and super-script D denote the contact between the curved-spoke leg and the surface profile.

**Figure 4:**
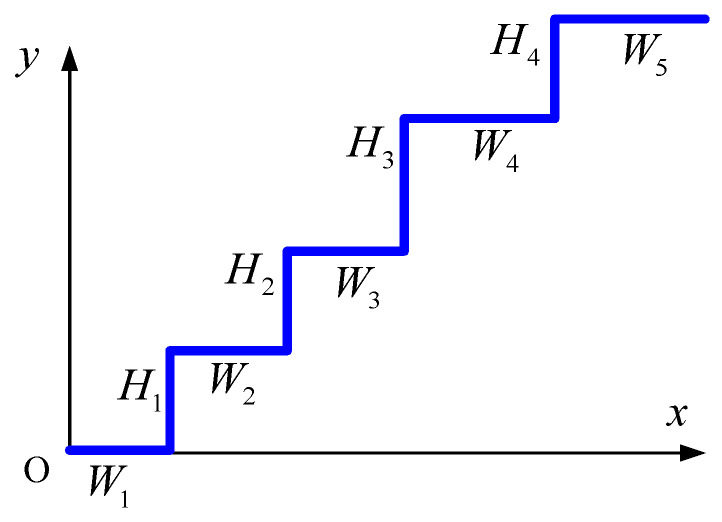
Surface profile function for an arbitrary stair geometry.

Next, we focused on the stair-climbing mechanism. To model a stair-climbing robot, the surface profile of a stair was defined as a continuous function, which can be expressed as follows:

(19) $$f=\sum_{i=1}^{N_{s}}\left(\frac{H_{i}}{2}\tanh\left(s_{a}\left(x-\sum_{k=1}^{i}W_{k}\right)\right)+\frac{H_{i}}{2}\right),$$

where $N_{s}$ is the number of steps, and $H_{i}$ and $W_{i}$ are the height and width of the i-th step, respectively, as shown in Figure 4. The corresponding normal and tangential unit vectors at contact node C can be expressed in terms of the surface profile function as

(20) $$n_{j}={\left.\left(-\frac{\partial f}{\partial x},1\right)/\sqrt{{\left(\frac{\partial f}{\partial x}\right)}^{2}+1}\right|}_{x=x_{j}^{C}},$$

(21) $$t_{j}={\left.\left(1,\frac{\partial f}{\partial x}\right)/\sqrt{{\left(\frac{\partial f}{\partial x}\right)}^{2}+1}\right|}_{x=x_{j}^{C}}.$$

**Figure 5:**
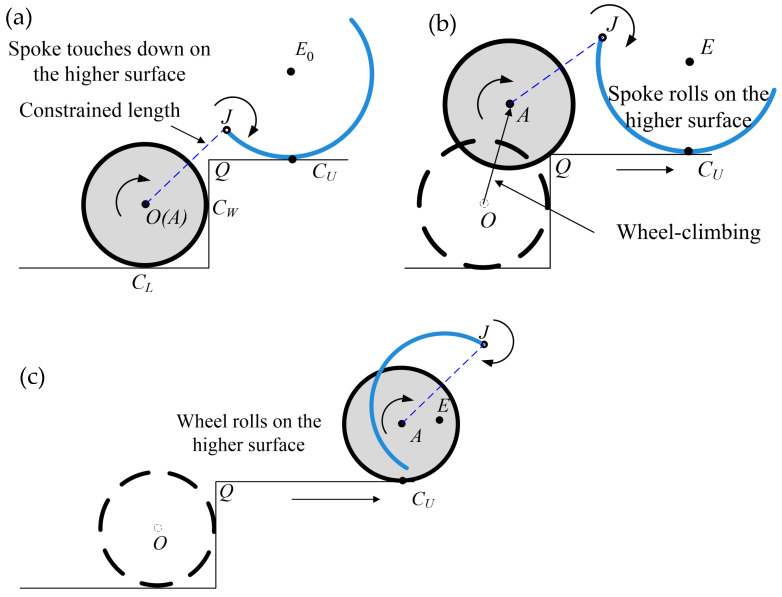
Scenario of single-wheel climbing locomotion; (**a**) phase 1: the tire rolls on the lower surface up to the wall of the stair and the spoke rotates and touches down on the higher surface; (**b**) phase 2: the wheel is elevated from the lower to the higher surface owing to the pulling of the spoke leg during spoke rolling; (**c**) phase 3: both the wheel and spoke leg roll on the higher surface.

Consequently, the four motor-driven tires with the rotating locomotion of curved-spoke legs can be simulated using Equations (1)–(20). Thus, the remaining and essential task is to determine the design and control parameters of the proposed locomotion and mechanism to enable the robot to climb the arbitrary stair geometry. For this, three phases of the locomotion scheme are proposed, as illustrated in Figure 5. After the motor-driven tire meets the wall of the stair at a contact point $C_{W}$, the curved-spoke leg rotates until it touches down on the contact point $C_{U}$ of the higher surface, as shown in phase 1 of Figure 5a. Owing to the constrained length between J and the wheel center, the rolling of the spoke leg around E in phase 2 of Figure 5b can produce a translation of the curved-spoke leg from left to right on the higher surface, resulting in the translation of the wheel from the lower to the higher surface, except in the case of an excessive slip between the spoke and the higher surface. To realize the proposed single-wheel climbing scenario, the following kinematic relations between the wheel, spoke leg, and stair geometry are introduced.

**Figure 6:**
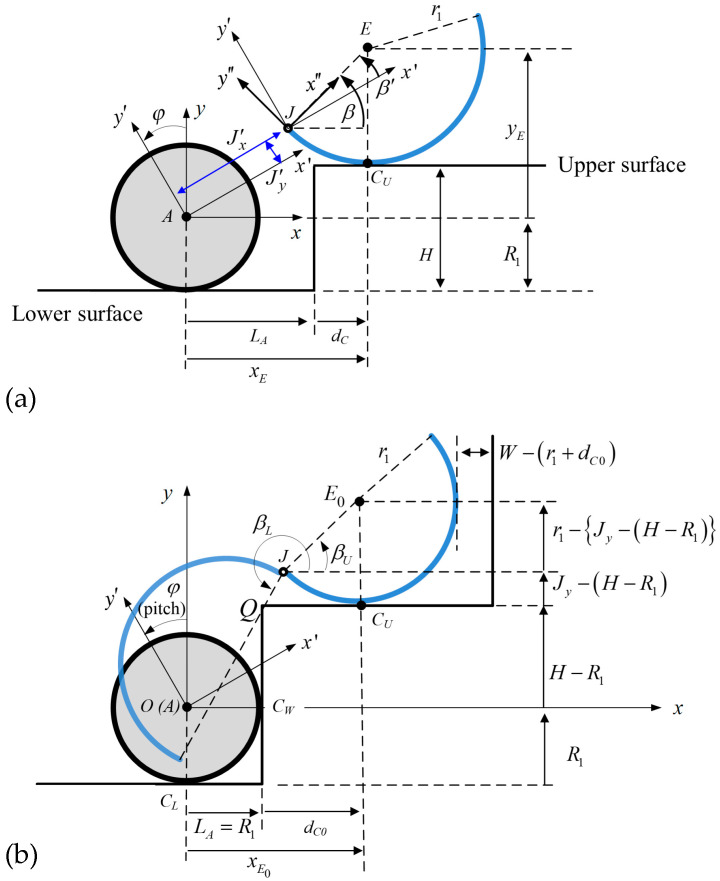
Configuration of the curved-spoke leg relative to the wheel, (**a**) the joint position J $\left(J_{x}^{\prime },J_{y}^{\prime }\right)$ and rotation angle β′ with respect to the body frame x’-y’-z’, and (**b**) the determination of joint position J for the success of the single-wheel climbing when the tire was in contact with the wall at $C_{W}$.

First, the relative motions of the wheel and spoke leg for the joint position J in Figure 5 are calculated from the following kinematic constraint as

(22) $$\left\|r_{J}-r_{A}\right\|\equiv l=\mathrm{const}.,$$

where the position vector $r_{J}$ is defined in terms of the rotation angle β′ of the curved-spoke leg in Figure 6a, corresponding to the configuration of phases 1, 2, and 3, which can be expressed as

(23) $$r_{J}=\left[\begin{array}{c} x_{E_{0}}-r_{1}\beta^{\prime }+r_{1}\cos\left(\beta_{U}+\pi +\beta^{\prime }\right)\\ y_{E_{0}}+r_{1}\sin\left(\beta_{U}+\pi +\beta^{\prime }\right) \end{array}\right],$$

and where the wheel center A lies on the three position candidates rolling on the stair surface, such that

(24) $$r_{A}=\left[\begin{array}{c} 0\\ u_{y} \end{array}\right],0\leq u_{y}\leq h-R_{1},$$

for vertical translation in phases 1 and 2, and

(25) $$r_{A}=r_{Q}+\left[\begin{array}{c} -R_{1}\cos\theta_{R}\\ R_{1}\sin\theta_{R} \end{array}\right],0\leq \theta_{R}\leq \pi /2,$$

for curving on edge Q in phase 2, and

(26) $$r_{A}=r_{Q}+\left[\begin{array}{c} u_{x}\\ R_{1} \end{array}\right],0\leq u_{x}\leq W-R_{1},$$

for rolling on a higher surface in phase 3, where

(27) $$r_{Q}=\left[\begin{array}{c} R_{1}\\ h-R_{1} \end{array}\right].$$

From the above kinematic equation with pure rolling, the transition from phase 1 to phase 3 in Figure 5 can be numerically calculated in the geometric design stage to examine the success of the single-wheel climbing. The position of the wheel pulled by the rolling curved-spoke leg toward the higher surface was evaluated with respect to the rolling angle β’ of the spoke. It was observed that the single-wheel climbing under the kinematic relation was performed by the pure rolling assumption. To validate its actual climbing operation, the dynamic equations considering the slip of the rolling wheels and spokes on the surface of stairs were also applied.

Second, kinematic inequality equations are proposed for the initial design of the curved-spoke geometry and its relative position $\left(J_{x}^{\prime },J_{y}^{\prime }\right)$ to the nearby wheel for the success of the single-wheel climbing using the rolling and pulling constraints of the curved-spoke leg. For this, the operating angle of the curved-spoke leg with respect to x′-y′-z′ (robot body-fixed) in Figure 6a is given as

(28) $$\beta^{\prime }=\beta -\varphi ,$$

where

(29) $$\beta =\tan^{-1}\left(\frac{y_{E}-J_{y}}{x_{E}-J_{x}}\right).$$

From Figure 6b, we measured the initial spoke angle $\beta_{U}$ when the wheel made contact with the wall and the spoke leg touched down on the higher surface in phase 1, denoting that

(30) $$\beta_{U}=\tan^{-1}\left(\frac{y_{E_{0}}-J_{y}}{x_{E_{0}}-J_{x}}\right),$$

where

(31) $$x_{E_{0}}=\sqrt{r_{1}^{2}-{\left\{r_{1}-\left(J_{y}-\left(H-R_{1}\right)\right)\right\}}^{2}}+J_{x}.$$

If $2r_{1}>J_{y}-\left(H-R_{1}\right)>0$ in Equation (31), then the real solution for the contact position can be derived; otherwise, there is no solution. This implies that the semicircle-like curved-spoke leg does not climb the stair if the height of the spoke joint is lower than that of the higher surface, that is, $J_{y}<\left(H-R_{1}\right)$ and $2r_{1}>J_{y}-\left(H-R_{1}\right)$.

In addition, the inequality $d_{C0}>d_{slip}>0$ in Figure 6b guarantees that the spoke leg can touch down on the higher surface and start rolling at $d_{C0}$ on the higher surface in phase 1. Because the slip of the curved-spoke leg during rolling on the higher surface in phase 2 may occur due to gravity, the margin $d_{slip}$ allowing the slip distance was applied to prevent the fall of the spoke leg from the higher surface. The inequality $W-r_{1}>d_{0}$ in Figure 6b prevents the blockage of the wall of the next step on the rolling spoke leg.

Consequently, the wheel-climbing requirement for phase 1 can be summarized as

(32) $$2r_{1}>J_{y}-\left(H-R_{1}\right)>0\;\mathrm{for}\;\varphi \in \left[0,\varphi_{\max}\right],$$

(33) $$W-r_{1}>d_{C0}>d_{slip}>0\;\mathrm{for}\;\varphi \in \left[0,\varphi_{\max}\right],$$

(34) $$0<d_{C0}<W-r_{1},$$

where

(35) $$\begin{array}{cc} d_{C0} & =x_{E0}-R_{1}\\ & =\sqrt{r_{1}^{2}-{\left\{r_{1}-\left(J_{y}-\left(H-R_{1}\right)\right)\right\}}^{2}}+J_{x}-R_{1} \end{array},$$

(36) $$\left[\begin{array}{c} J_{x}\\ J_{y} \end{array}\right]=\left[\begin{array}{c} J_{x}^{\prime }\cos\varphi -J_{y}^{\prime }\sin\varphi \\ J_{x}^{\prime }\sin\varphi +J_{y}^{\prime }\cos\varphi \end{array}\right],$$

and where the maximum body pitch angle is assumed to be the slope of the stair $\varphi_{\max}=\tan^{-1}H/W$, and $d_{slip}$ is the margin allowing for the slip distance of the curved-spoke leg on the surface of a stair.

**Figure 7:**
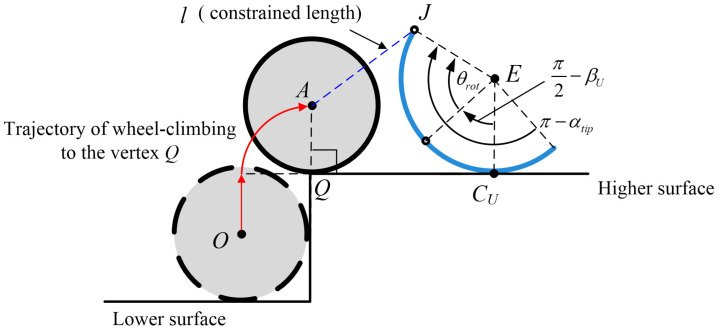
Configuration of the kinematic inequality condition for wheel climbing to vertex Q along with the trajectory of translational motion; the spoke rotates with $\theta_{rot}=\beta_{U}-\beta$ while maintaining the constrained length l and pulling the wheel.

The wheel-climbing requirement for phase 2 may be also proposed as an arc length of the spoke leg longer than that required for bringing the wheel to the vertex Q, as illustrated in Figure 7

(37) $$\pi -\alpha_{tip}>\theta_{rot}+\frac{\pi }{2}-\beta_{U}\mathrm{for}\;\varphi \in \left[0,\varphi_{\max}\right],$$

subject to

(38) $$\left\|r_{J}-\left(r_{Q}+\left[\begin{array}{c} 0\\ R_{1} \end{array}\right]\right)\right\|=l,$$

where $r_{J}$ is the function of the angle $\theta_{rot}=\beta_{U}-\beta$ in Equation (23) and $\left(J_{x}^{\prime },J_{y}^{\prime }\right)$, and this inequality equation is numerically calculated in terms of $\left(J_{x}^{\prime },J_{y}^{\prime }\right)$.

The four kinematic inequality Equations (32)–(34) and (37) may provide the design requirement for the geometry of the wheel and curved-spoke leg and the relative position for the success of single-wheel climbing on the prescribed stair geometry. It should be noted that the kinematic inequality conditions are derived from the geometric relations under no-slip conditions. Therefore, this kinematic design configuration for single-wheel climbing was validated in the next section through the dynamic equations of motion considering frictional slip.

**Figure 8:**
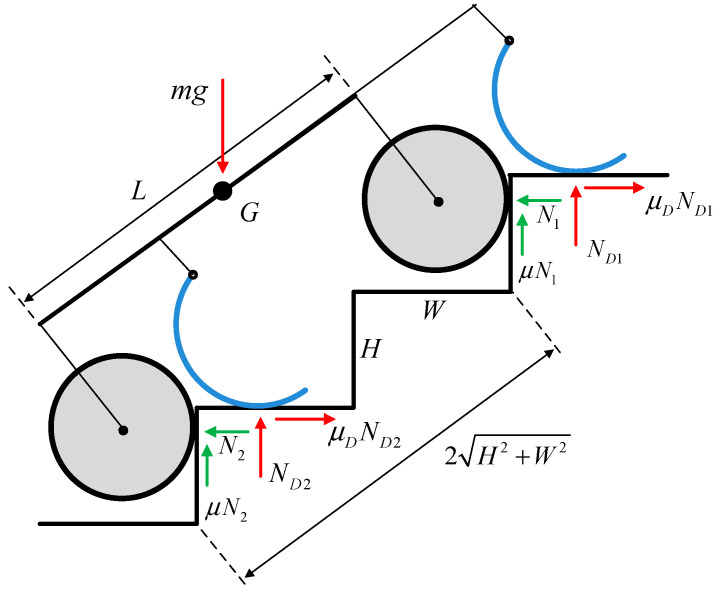
Static force diagram during stair climbing if the wheelbase L is approximated to the hypotenuse of several steps; $L\approx 2\sqrt{H^{2}+W^{2}}$.

This conceptual robot aims to enable stair climbing with a zero required minimum friction coefficient. It can climb a stair as a legged robot if the curved-spoke leg is well-defined under the kinematic inequality conditions in Equations (32)–(34) and (37), and the wheelbase is adjusted to nearly the hypotenuse of several steps. In this case, the wheel climbing of the front and rear axles was synchronized, as illustrated in Figure 8. If the static friction coefficient is $\mu_{s}\equiv \mu =\mu_{D}$, the balance equation for the static state can expressed as

(39) $$N_{D1}+N_{D2}=\frac{mg}{1+\mu_{s}^{2}},$$

where the $N_{D1}$ and $N_{D2}$ of the spoke legs can be produced by the joint torque of the spokes. This implies that the static state in Equation (39) holds even if the static friction coefficient $\mu_{s}$ is zero; thus, the required minimum friction coefficient turns out to be zero, as in the static analysis of the wheeled or rocker–bogie stair-climbing robots [38]. It should be noted that legged robots typically have zero required minimum friction coefficients because their legs hop rather than roll during stair climbing. Therefore, the proposed mechanism can also be categorized as a stair-climbing legged robot, given that wheels are additionally attached to the legged robot for flat road driving.

## 3. Results

A well-defined kinematic configuration between the wheel and curved-spoke leg is critical for the successful stair climbing of the proposed robot. The kinematic inequality conditions of wheel climbing are the necessary conditions for the single wheel of the robot to climb stairs. First, the parametric boundary for satisfying the kinematic inequality conditions was determined to determine the system’s geometric configuration for successful single-wheel climbing. After the specific design parameters were selected, the stair-climbing simulation based on the dynamic model was conducted to validate the stair-climbing performance of the whole robot.

### 3.1. Kinematic Results for the Single-Wheel Climbing

In the subsequent analysis, we focused on the kinematic model for a single wheel to climb a stair with the aid of the locomotion of a curved-spoke leg, and the configuration of the kinematic model is described in Figure 6 and Figure 7. The system parameters of the robot, which are listed in Table 1, are applied to the single wheel and its nearby curved-spoke leg. The friction coefficient was set to 0.3 for this simulation, which is relatively low for tire dynamics. Nevertheless, the simulation results demonstrated the stair-climbing ability of the single wheel at a low friction coefficient, as explained in the required minimum friction coefficient of the static analysis in Figure 8. The stair geometry, such as the width and height of the stair, was assumed to be constant in the simulation.

**Figure 9:**
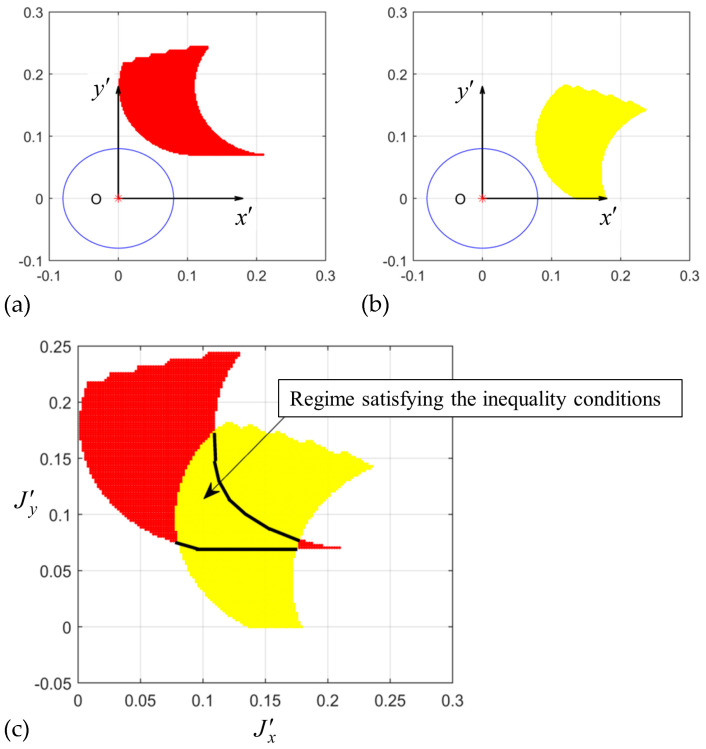
The regime of joint positions of the spoke leg relative to the wheel satisfying the kinematic inequality conditions at (**a**) $\varphi =0^{\circ }$ and (**b**) $\varphi =\tan^{-1}H/W=31^{\circ }$, and the (**c**) regime satisfying the inequality conditions for both pitch angles.

To determine the kinematic inequality conditions, the geometric boundary of the spoke joint position satisfying the inequality conditions was calculated. The spoke joint position relative to a wheel in the body-fixed frame is important for the spoke leg to successfully roll onto the higher surface and bring the nearby wheel to the surface. We observed that the pitch angle of the robot changed when it climbed onto the slope of the stair. Therefore, the joint position for stair climbing in the body-fixed frame should differ from that in the ground-reference frame. For a fixed slope of a stair to be climbed, the joint position should satisfy the kinematic inequality conditions of the robot driving on both the flat ground and a slant stair. Figure 9a shows the joint position requirement to satisfy the kinematic inequality conditions when the front wheel climbs the first step at the zero-pitch angle. As the robot drives onto the slant of the stair for steady-state climbing, the body pitch changes to the slope of the stair, and the joint position requirement relative to the wheel also changes, as illustrated in Figure 9b. The intersection of the two requirements was selected as the joint position candidate relative to the wheel in the body-fixed frame, as shown in Figure 9c.

**Figure 10:**
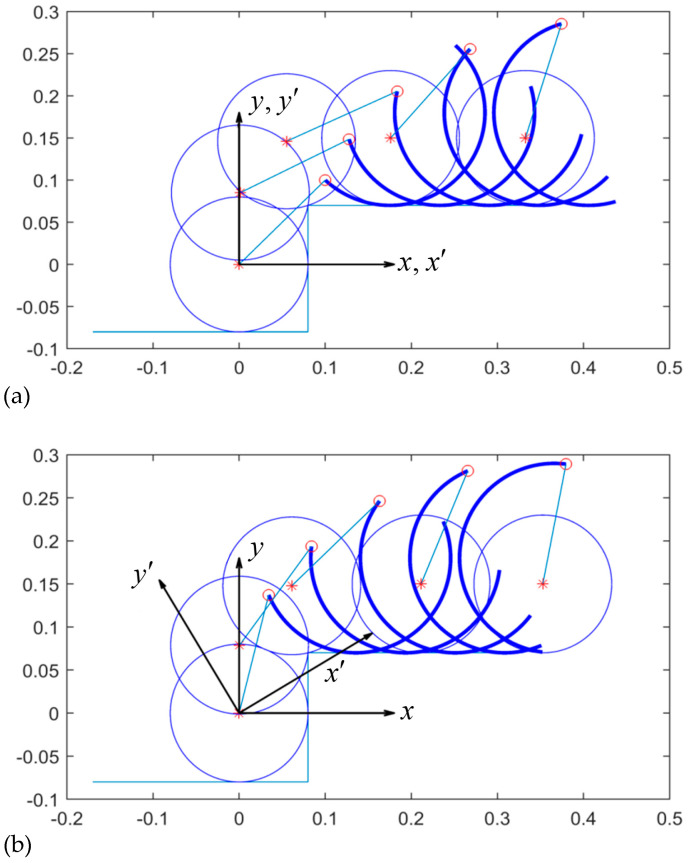
Kinematic sequential motion of wheel-climbing for $\left(J_{x}^{\prime },J_{y}^{\prime }\right)=\left(0.1,0.1\right)$ at (**a**) $\varphi =0^{\circ }$ (horizontal body) and (**b**) $\varphi =\tan^{-1}H/W=31^{\circ }$ (slant body).

In the subsequent analysis, the joint position at the intersection of the two requirements was chosen to be $\left(J_{x}^{\prime },J_{y}^{\prime }\right)=\left(0.1,0.1\right)$ in the body-fixed frame, as in the case of the joint position satisfying the two requirements. Figure 10a demonstrates that the wheel at the zero-pitch angle can be translated up to the vertex Q of the stair as the curved-spoke leg rolls on the higher surface. Figure 10b shows that the wheel can also kinematically climb up to the vertex Q in the slant body case of $\varphi =\tan^{-1}H/W=31^{\circ }$.

### 3.2. Dynamic Results for the Stair Climbing of the Whole Robot

In this section, the dynamic response of the stair climbing of the whole robot was estimated through dynamic equations of motion, as expressed in Equations (1) and (2). For the dynamic simulation of the whole system, the front and rear axles were included. Therefore, the stair climbing of the system required the simultaneous climbing of the front and rear wheels with the aid of the locomotion of both the front and rear curved-spoke legs. One important parameter for the synchronization of the front and rear wheel-climbing was the wheelbase, which is the distance between the front and rear axles. In reference [1], numerous stair-sensing techniques for stair climbing were reviewed. Using LiDAR, stereo cameras, proximity sensors, and other technologies, the size of the stairs can be measured in real-time. When the hypotenuse of two steps is measured, it is assumed that a variable wheelbase mechanism is equipped and controlled to adjust before encountering the next two steps. The wheelbase was assumed to be the hypotenuse of two steps. The speed of the curved-spoke legs was set to be linearly varied with the slope $\gamma =\left(\omega_{f}-\omega_{o}\right)/\varepsilon$ within a certain range ε of rotation angles to reduce chattering and impact.

(40) $$\dot{\beta }=\gamma \left(\beta -\beta_{L}\right)-\omega_{o}\;\mathrm{or}\;\dot{\beta }=-\gamma \left(\beta -\beta_{U}\right)-\omega_{o}.$$

**Figure 11:**
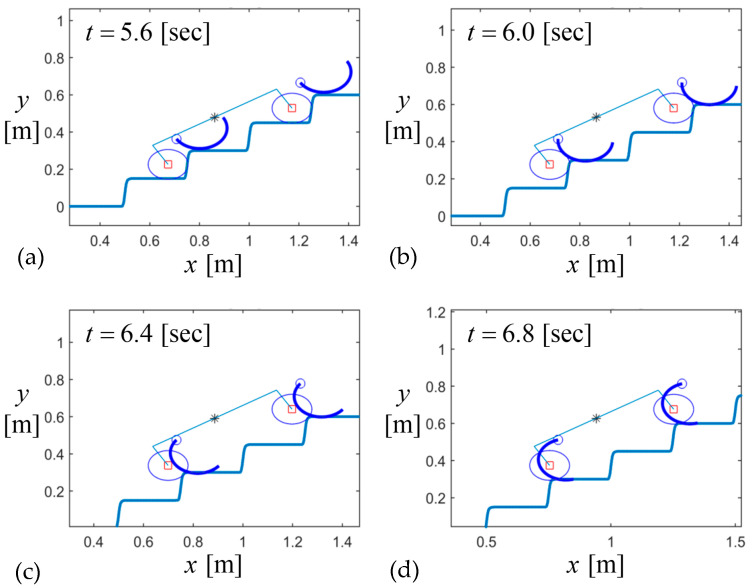
Locomotion of the robot during stair climbing for $\left(J_{x}^{\prime },J_{y}^{\prime }\right)=\left(0.1,0.1\right)$ and $L=2\sqrt{H^{2}+W^{2}}$=0.58: (**a**) phase 1, (**b**) initial phase 2, (**c**) mid-phase 2, and (**d**) final phase 2.

Figure 11 shows the dynamic sequence of the stair climbing of the whole body. As the wheelbase was set to be the hypotenuse of several steps, the sequential motion of the front and rear wheels and their curved-spoke legs was synchronized and similar to the kinematic sequence of the single wheel in Figure 10.

**Figure 12:**
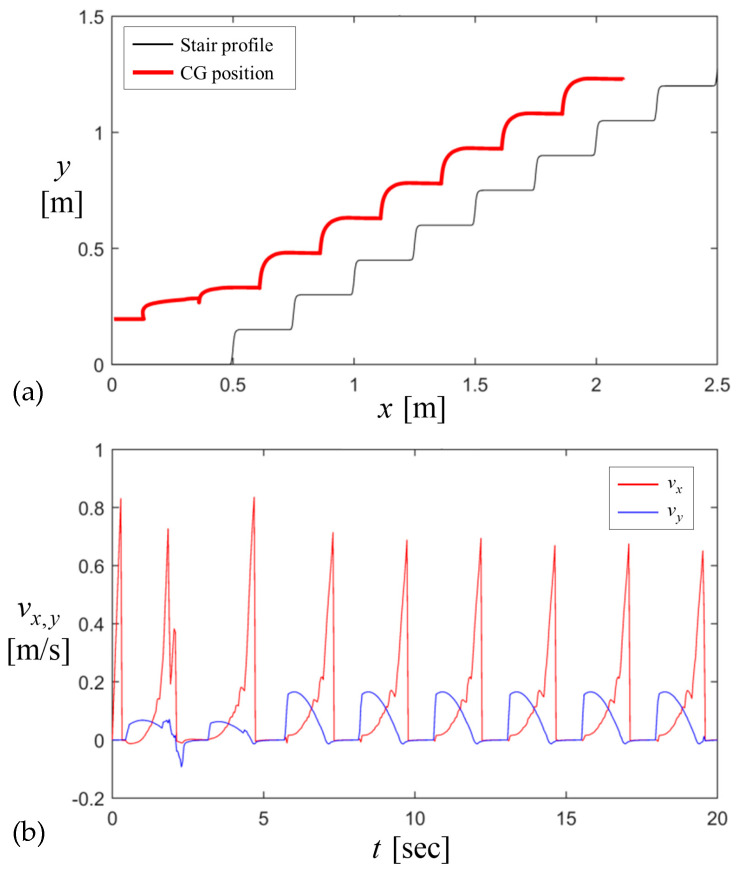
Stair-climbing response of the dynamic model for $\left(J_{x}^{\prime },J_{y}^{\prime }\right)=\left(0.1,0.1\right)$ and $L=2\sqrt{H^{2}+W^{2}}$=0.58: (**a**) the CG position and (**b**) velocity; wheelbase L is assumed to be adjusted to the length of the hypotenuse of 2 steps.

The dynamic trajectory of the CG was traced during stair climbing as illustrated in Figure 12a. After the robot climbs the first step, its trajectory during the steady state climbing oscillates as a cycloid according to the periodic profile of the stair. The corresponding velocity of CG varies periodically in Figure 12b as the stair climbing acts as the periodic motion. When the robot meets the wall of each step, the horizontal speed drops to zero. As the wheel climbs each step, the vertical speed increases to a certain value but drops back to zero as the robot drives on the flat surface of each step.

**Figure 13:**
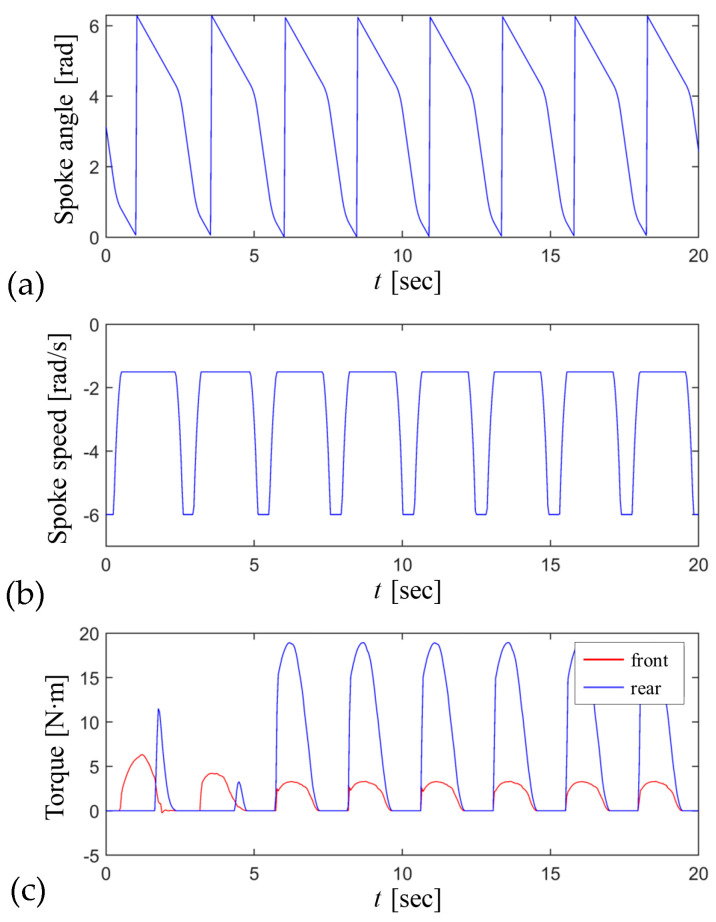
Input values of the front and rear curved-spoke legs for stair climbing at $\left(J_{x}^{\prime },J_{y}^{\prime }\right)=\left(0.1,0.1\right)$ and $L=0.58=2\sqrt{H^{2}+W^{2}}$: (**a**) the spoke angle, (**b**) spoke speed, and (**c**) spoke torque.

The rotating angle and speed of the curved-spoke leg were controlled by the pre-defined speed profile in Equation (40). The rotating speed of the spoke leg increased when the spoke leg rotated freely in the air, but decreased as the spoke leg rolled onto the higher flat surface of each step. Figure 13a,b show the variations per one revolution of the rotating angle and speed of the spoke leg during stair climbing. The joint torques of the front and rear spoke legs required to climb each step are also demonstrated in Figure 13c. With an increase in the friction coefficient, the required joint torque for stair climbing decreased, because the wheel traction on the wall of the stairs reduced the normal loads of the curved spoke legs on the higher surface.

## 4. Discussion

This study proposed a simple wheel-leg mechanism for stair climbing, as well as flat road driving. Stair climbing was achieved using the locomotion of the motor-driven tires and curved-spoke legs. Particularly, the design configuration requirements of the motor-driven tire and curved-spoke leg for various stair geometries were proposed. Thereafter, the stair-climbing ability was validated through a dynamic model that allowed for frictional slips. The following conclusions were drawn from these results:

− Wheel climbing can be achieved by utilizing a curved-spoke leg.
− Wheel climbing is a necessary condition for the stair climbing of the whole robot.
− The spoke position relative to a wheel should be carefully determined for both the flat and slant robot using the proposed kinematic inequality constraints.
− The proposed robot could effectively climb a given stair geometry if the curved-spoke leg is well defined under the kinematic inequality conditions, and the wheelbases are adjusted to the hypotenuse of several steps.
− The required minimum friction coefficient for the static state of stair climbing is very low for wheel-spoke leg locomotion if the kinematic wheel-climbing conditions are met.

## Figures and Tables

**Table 1:** System parameters of stair-climbing robot.

Symbol	Value	Unit
*m*	30	kg
*R*	0.08	m
*r*	0.11	m
*H*	0.15	m
*W*	0.25	m
$k_{c}$	1.0 × 10^6^	N/m^2^
$k_{D}$	1.0 × 10^6^	N/m^2^
$C_{c}$	2.5 × 10^5^	Ns/m^2^
$C_{D}$	2.5 × 10^5^	Ns/m^2^
μ	0.3	-
$\mu_{D}$	0.3	-
